# Upcycling imperfect broccoli and carrots into healthy snacks using an innovative 3D food printing approach

**DOI:** 10.1002/fsn3.3820

**Published:** 2023-11-13

**Authors:** Safoura Ahmadzadeh, Taylor Clary, Alex Rosales, Ali Ubeyitogullari

**Affiliations:** ^1^ Department of Food Science University of Arkansas Fayetteville Arkansas USA; ^2^ Department of Chemical Engineering University of California Berkeley California USA; ^3^ Department of Biological and Agricultural Engineering University of Arkansas Fayetteville Arkansas USA

**Keywords:** 3D food printing, broccoli, carrot, food loss and waste, imperfect vegetables, snacks

## Abstract

Vegetables are healthy foods with nutritional benefits; however, nearly one‐third of the world's vegetables are lost each year, and some of the losses happen due to the imperfect shape of the vegetables. In this study, imperfect vegetables (i.e., broccoli and carrots) were upcycled into freeze‐dried powders to improve their shelf‐life before they were formed into food inks for 3D printing. The rheology of the food inks, color analysis of the uncooked and cooked designs, and texture analysis of the cooked designs were determined. The inks with 50% and 75% vegetables provided the best printability and shape fidelity. 3D printing at these conditions retained a volume comparable to the digital file (14.4 and 14.3 cm^3^ vs. 14.6 cm^3^, respectively). The control, a wheat flour‐based formulation, showed the lowest level of stability after 3D printing. The viscosity results showed that all the food inks displayed shear‐thinning behavior, with broccoli having the greatest effect on viscosity. There was a significant color difference between uncooked and cooked samples, as well as between different formulations. The hardness of the baked 3D‐printed samples was affected by the type and content of vegetable powders, where carrot‐based snacks were notably harder than snacks containing broccoli. Overall, the results show that 3D food printing can be potentially used to reduce the loss and waste of imperfect vegetables.

## Graphical abstract

Although vegetables provide numerous health benefits, they are lost and wasted at a very high rate (i.e., about one‐third of the total production), with some being due to their imperfect shape. This study aimed to upcycle imperfect broccoli and carrots using an innovative approach based on 3D food printing to generate nutritious, healthy snacks. The results revealed that a 75% carrot/broccoli to 25% flour ratio produced the best shape fidelity and volume, comparable to the 3D digital model.

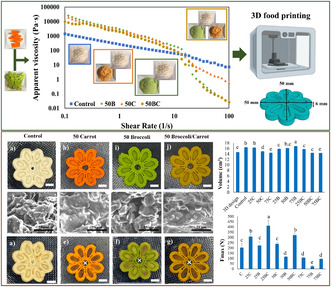

## 1 INTRODUCTION

As the need for enough food to sustain the growing population increases, the amount of waste that is generated also increases. According to a 2021 Food and Agricultural Organization research report by the United Nations, one‐third of the food produced is wasted every year (United Nations, 2021). Compared to other foods, fruits and vegetables are wasted even at higher rates, ranging from 37% to 55% globally (FAO, 2011). This amounts to about 1.3 billion tons of fruit and vegetable waste annually (United Nations, 2021). Many fruits and vegetables are left on the field or tossed in processing because the produce is “ugly or imperfect.” The “ugly” produce is thrown out for cosmetic reasons, ranging from color and size to odd shapes and blemishes on the peel. These fruits and vegetables have the same nutritional values as the accepted fruits and vegetables but never make it to the market because of their poor appearance. After undergoing some processing, the imperfect produce, specifically vegetables in this case, can be upcycled using 3D food printing technology. Imperfect vegetables can be utilized as an alternative ingredient in 3D‐printed foods if the final food ink meets acceptable extrusion rheological parameters.

3D printing, a way of additive manufacturing, also referred to as digital fabrication technology, is a quickly emerging technology with many capabilities. The adoption of this technology poses many advantages, such as increased production speed, lower production costs, customer customization, decreased need for global transport, and decreased distribution time and cost (Shahrubudin et al., 2019).

Extrusion‐based 3D printing is currently the most applicable approach for the food industry. The food “inks” are extruded through a nozzle in layers to form a 3D food product. There are three different potential forces used for extrusion‐based technologies, including screw, piston, or compressed air. The extrusion‐based 3D printing technology has been shown to work for a wide range of food materials, including chocolate, custard creams, pastes, sugar candies, starch, and alginate‐pectin (Ahmadzadeh & Ubeyitogullari, 2022, 2023; Outrequin et al., 2023; Rysenaer et al., 2023). The extrusion technique uses a robotic arm that moves along a surface with a cylindrical syringe that dispenses the food paste. There are certain parameters that must be considered when developing the food paste, such as the capability of the food paste to be extruded through the nozzle, the ability for the food paste to have a sufficient viscosity for the layers to stack without defects, and the resolution of the final product due to the stability and definition of the food paste (Le‐Bail et al., 2020).

3D food printing technology has made its way into the food industry with the capability of impacting manufacturing processes. The technology poses advantages for individualized nutrition, raw material utilization, and customizable product design (Ahmadzadeh et al., 2023; Shen et al., 2023; Wu et al., 2023). Some of the notable capabilities of 3D food printing include personalization, on‐demand production, and, as highlighted in this research, reduction of food loss and waste (Derossi et al., 2021). 3D printing can bring products to the customer, which reduces the reliance on shipping, packaging, and distribution, alleviating the food supply chain of immense pressure (Shahrubudin et al., 2019). In addition, the increased attractiveness of printed foods could reduce the resistance of children and other age groups to consume certain foods. This caters to the idea that the breakdown of a food product that is unappealing to the eye and then rebuilt to improve consumer attractiveness and acceptance.

Previously, freeze‐dried mango powder, in addition to flour, water, and olive oil, was used to develop a dough formulation. The goal of that research was to determine the optimum ratio of ingredients for printability (Liu et al., 2019). Derossi et al. (2020) aimed to study the porosity fraction of a similar dough filament and the ability to develop cereal snacks where the formulation was optimized for ideal extrusion‐based printing (Derossi et al., 2020). Food materials are categorized as natively printable, traditionally non‐printable, and alternative (Pulatsu & Lin, 2021). The alternative category is ingredients that become more palatable and attractive after undergoing 3D food printing, such as insect powders and algal components. Vegetables are traditionally considered non‐printable materials, considering their high moisture content and lack of suitable mechanisms, such as gelation, agglomeration, or solidification (Pulatsu & Lin, 2021). However, if the vegetable's moisture content is decreased and combined with an ingredient capable of agglomerating, it becomes part of the alternative category. Even though several studies have investigated the 3D printability of various food components, upcycling imperfect vegetables via 3D food printing has not been fully explored. Among vegetables, carrots and broccoli are especially lost or wasted to a greater extent (FAO, 2011, 2018; Melini et al., 2020).

Therefore, the objective of this study was to determine the optimal ratio of imperfect carrot and broccoli for ideal 3D food printing. Specific objectives were to (i) optimize the 3D printing conditions for different carrot/broccoli ratios, (ii) characterize the properties of the food inks by analyzing their viscosity, and (iii) determine the printability, color, microstructure, and texture of the 3D‐printed snacks. This study aimed to develop a snack cracker that utilizes imperfect vegetables as alternative ingredients that add value both nutritionally and economically.

## 2 MATERIALS AND METHODS

### 2.1 Materials

Imperfect carrots and broccoli were provided by Taylor Farms. All‐purpose wheat flour (with 76.7% (w/w) carbohydrate and 10.0% (w/w) protein contents based on the manufacturer's specifications), extra virgin olive oil, and sodium chloride were all obtained from a local grocery store.

### 2.2 Vegetable powder preparation

The imperfect vegetables were refrigerated for no more than 2 days before their processing steps began. Before blanching, the vegetables were sliced into small pieces (~4 cm in dimension). The vegetables were then steam‐blanched in a steamer (Dixie, M‐6 Steam Blancher‐Cooler) at 90°C for 3 min to inactivate peroxidase (Kidmose & Martens, 1999). After blanching, the vegetables were packed into Ziploc bags and frozen for at least 24 h. Next, the samples were freeze‐dried at −45°C and 7.3 Pa (LABCONCO). The produce was held at these conditions for 48 h.

The dried produce was then milled into a fine powder with a Blizer 2 food processor (Robot Coupe USA Inc.). The fine powder was then placed in 30‐ to 50‐g increments in a Meinzer II sieve shaker (Advantech, OH, USA) for about 15 min or until most of the powder had passed through. A 60‐mesh sieve with 250 μm openings was used to separate out the larger particles. The powder with particle size ≤250 μm was then placed into a Ziploc bag and stored in a refrigerator (4°C) until further use.

### 2.3 3D printing of product formulation

#### 2.3.1 Paste formation

Table 1 includes the amounts of vegetable powder, flour, salt, and olive oil that were included in each paste (Derossi et al., 2020). Once all ingredients had been added to an empty beaker, they were mixed before water was added to the desired consistency. The amount of water was adjusted between 25 and 50 mL based on the vegetable ratio to achieve the required consistency. The samples were mixed by hand with a spatula until the sample was a homogenous paste. At this point, the paste was ready for printing, color analysis, and rheology.

**TABLE 1:** Ink formulations used for 3D printing.

	Abbreviation	Wheat flour (g)	Carrot (g)	Broccoli (g)	Salt (g)	Olive oil (g)
Control	Control	32	–	–	0.5	3
25% carrot	25C	24	8	–	0.5	3
25% broccoli	25B	24	–	8	0.5	3
25% mix	25BC	24	4	4	0.5	3
50% carrot	50C	16	16	–	0.5	3
50% broccoli	50B	16	–	16	0.5	3
50% mix	50BC	16	8	8	0.5	3
75% carrot	75C	8	24	–	0.5	3
75% broccoli	75B	8	–	24	0.5	3
75% mix	75BC	8	12	12	0.5	3

#### 2.3.2 3D food printing

**FIGURE 1:**
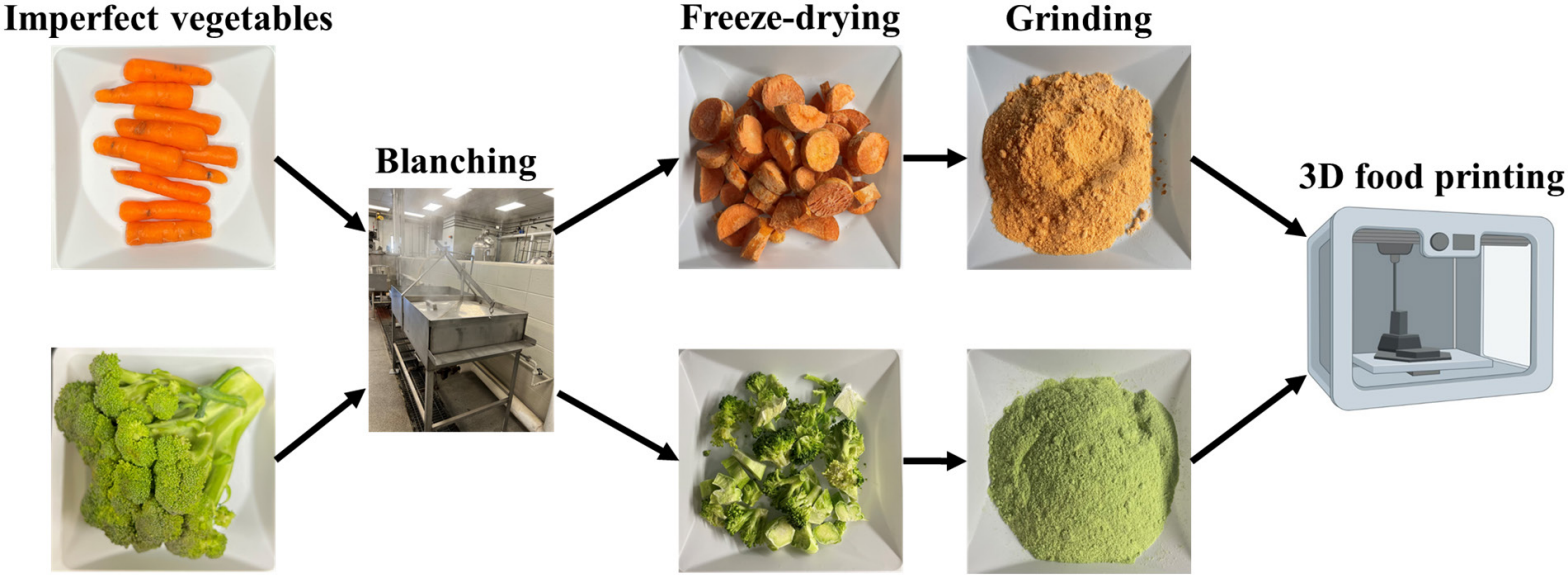
Process diagram of 3D printing of imperfect broccoli and carrot formulations.

**FIGURE 2:**
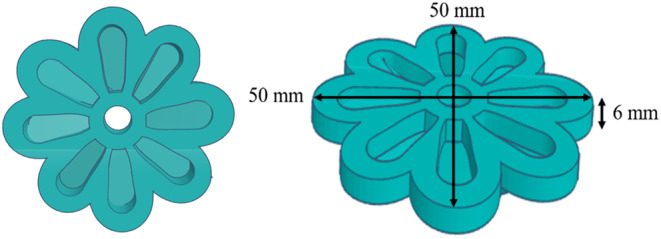
The 3D model used for printing.

An extrusion‐based 3D food printer (Foodini, Natural Machines, Spain) was used to print the 3D food products. A 1.5‐mm nozzle was utilized to print a flower shape with a height of 6 mm and 4 layers. The geometry was 3D‐printed a total of six times. Figure 1 shows the 3D printing process. The printing parameters, such as print speed and extrusion rate, were selected for the best printability. The printability was assessed by comparing the dimensions of the prints to those of the digital model (Figure 2).

#### 2.3.3 Post‐printing processing

The 3D‐printed samples were baked in a smart oven with pure light technology (Brava, CA, USA) at 177°C for approximately 8 min depending on the sample.

### 2.4 Viscosity measurement

A controlled‐stress rheometer (AR 2000 Rheometer; TA Instruments) was used to determine the viscosity of the samples. The rheometer was calibrated for inertia before each use, as well as the gap height with the attachment. A 40‐mm steel sand‐blasted attachment was utilized. The range for shear rate was set at 0.1–100 1/s, and the measurements were recorded at 25°C.

### 2.5 Macroscopic and microstructural properties

The images acquired after 3D printing were compared to the digital 3D model to evaluate the printing accuracy. The volume of the printed samples was also measured and compared to the volume of the 3D model. A ruler was used to establish the scale bar in the photographs.

The microstructure of the snacks printed with a 50% vegetable ratio and the control sample was investigated using an FEI NovaNanolab200 Dual‐Beam system equipped with a 30‐kV SEM FEG column and a 30‐kV FIB column (FEI Company). Thin cross sections of freeze‐dried 3D‐printed samples were coated with a gold layer using a sputter‐coater (EMITECH SC7620 Sputter Coater). Finally, SEM imaging was performed at a 15 kV acceleration voltage and a current of 10 mA.

### 2.6 Color analysis

The color of the samples was determined using a colorimeter (Minolta CR‐300, Konica Minolta). The colorimeter was calibrated with a white tile (L* = 97.12, a* = +5.25, b* = −3.49) provided with the equipment before each use. The color of the samples was measured before and after baking, where L*, a*, and b* were recorded. A total of six readings were carried out for each food ink, and the results were reported as mean ± standard deviation.

### 2.7 Texture analysis

The cooked samples were analyzed for their texture using a TA‐XT2i Texture Analyzer equipped with Exponent software (Stable Micro Systems, Ltd.). The hardness of the cooked 3D‐printed samples was determined (Jia et al., 2020). A 5‐kg maximum load cell was used to calibrate force before the experiments. The clearance between the flat compression plate and the base was set at 60 mm. A cylindrical probe with a diameter of 4 cm was used for the compression. The cooked samples were compressed to a 50% strain with a pre‐test speed of 1.5 mm/s, a test speed of 1.0 mm/s, and a post‐test speed of 1.0 mm/s.

### 2.8 Statistical analysis

Statistical analysis was conducted using SPSS Statistics software. The color and texture data were analyzed using a one‐way ANOVA with Tukey's multiple comparison test at a significance level of 0.05.

## 3 RESULTS AND DISCUSSION

### 3.1 Printability

**FIGURE 3:**
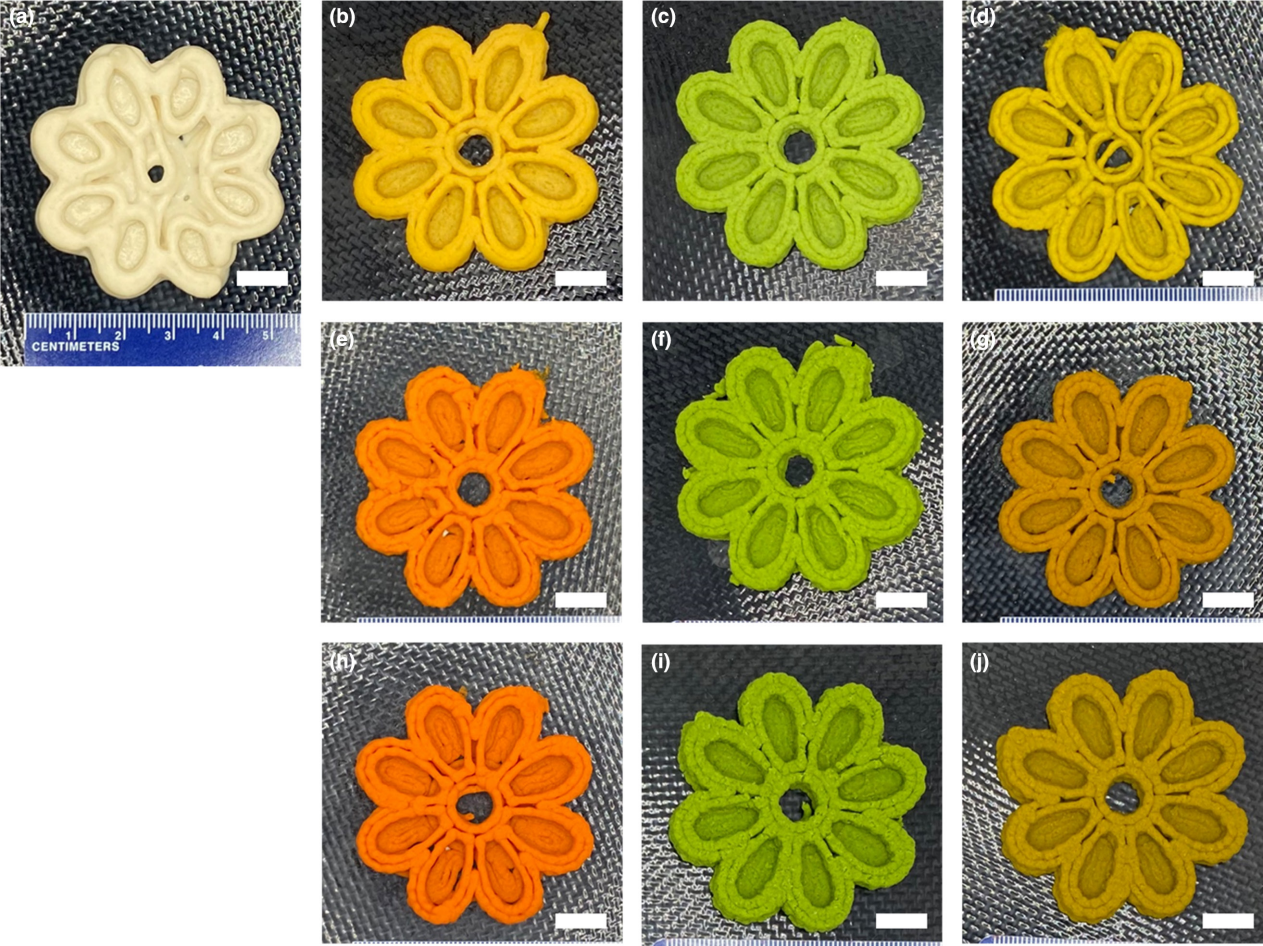
3D‐printed uncooked products of each food ink formulation (a: control; b: 25 carrot:75 flour; c: 25 broccoli:75 flour; d: 25 broccoli/carrot:75 flour; e: 50 carrot:50 flour; f: 50 broccoli:50 flour; g: 50 broccoli/carrot:50 flour; h: 75 carrot:25 flour; i: 75 broccoli:25 flour; j: 75 carrot/broccoli:25 flour, mass ratios). The scale bars in all images represent 1 cm.

**FIGURE 4:**
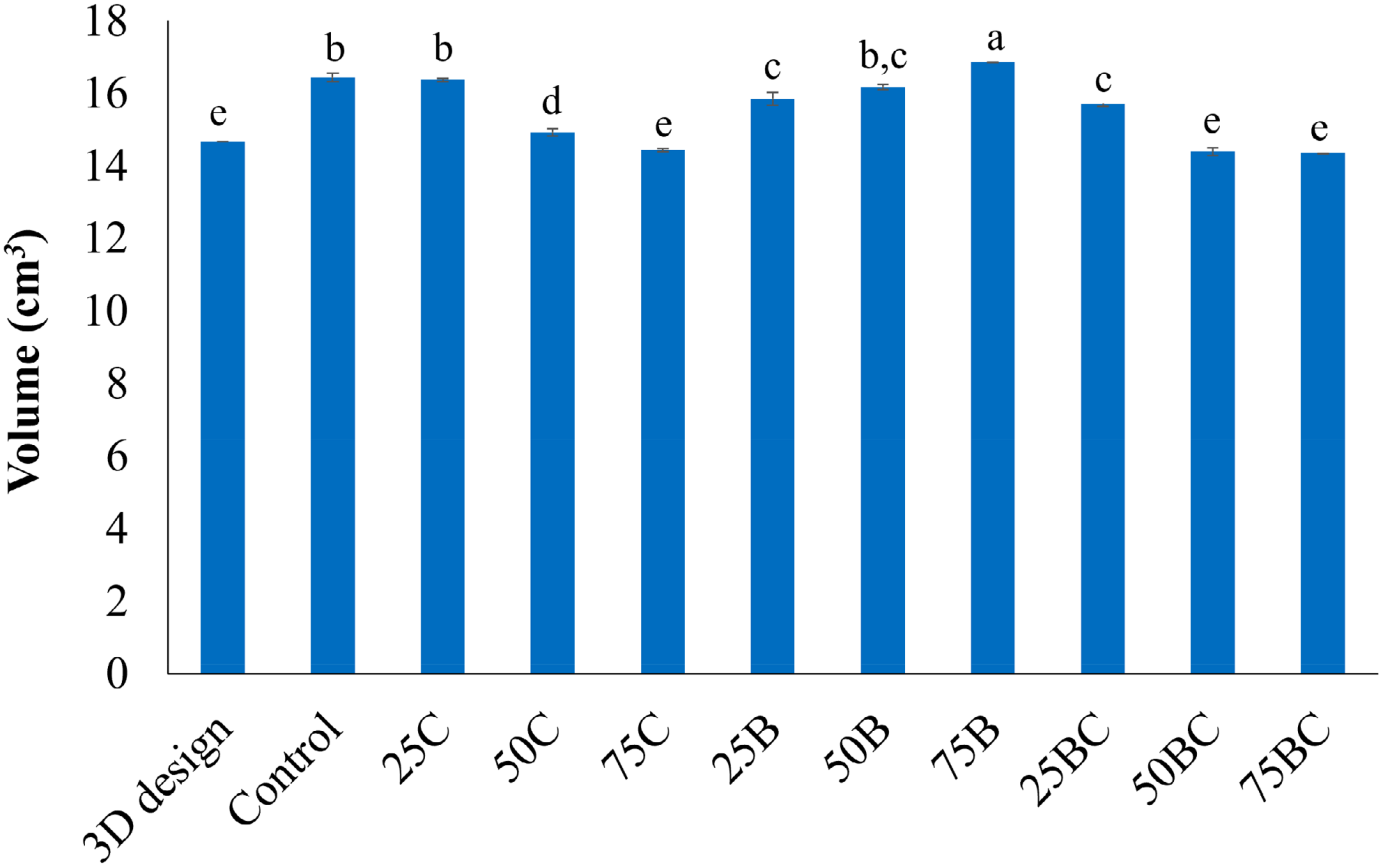
The volume of the digital 3D geometry and 3D‐printed samples. Means that do not share a letter are significantly different (*p* < .05).

On the basis described in a similar formulation (Derossi et al., 2020), broccoli and carrot powders were added in various amounts as a substitute for wheat flour. Wheat flour was not completely substituted due to the need for an agglomerating ingredient because vegetable powders serve best as an alternative ingredient. The food inks developed from Table 1 appeared to have similar characteristics as cookie or bread doughs. The settings for the printing were determined based on the preliminary 3D printing experiments at different carrot/broccoli formulations. For successful 3D printing, it is critical to keep the ink homogenous and eliminate any air pockets in the cartridge. To evaluate printing accuracy, the geometric features of the printed objects, including volume, were assessed using digital image analysis and compared to those of the digital 3D geometry. Figure 3 depicts images of 3D‐printed objects with varying ratios of wheat flour, carrot, and broccoli. In addition to visually evaluating shape accuracy and resolution, the volumes of the 3D‐printed samples were estimated and compared to the volume of the digital 3D geometry (Figure 4). Higher and lower volumes in comparison to the volume of the 3D design suggested a lower build quality. The printing performance of the flour dough was inadequate (Figure 3a), as evidenced by the significantly low resolution and the inability to maintain shape in the matrix that was printed without incorporating vegetables, which was probably due to the viscoelastic gluten network (Masbernat et al., 2021). The printed object had a good level of shape retention when 75% carrot and broccoli (75BC) was used (Figure 4). However, the physical stability of the broccoli‐flour samples extruded through the nozzle was lower than that of the carrot‐containing counterpart samples, resulting in larger volumes of the 3D‐printed products compared to the digital 3D geometry. This observation highlighted the significance of matrix strength in 3D printing. By combining carrot and broccoli with wheat flour, the dimensional stability was significantly improved when 75% or 50% vegetable formulation was employed (*p* < .05). However, when a lower vegetable ratio (25%) was used, the paste's structure prevented the formation of a good shape after printing, as evidenced by the noticeable lines observed after printing (Figure 3d). 3D printing of 50% and 75% carrot/broccoli‐flour formulations yielded the best results (volumes of 14.4 and 14.3 cm^3^, respectively) with the fewest geometrical errors and volumes comparable to the digital file (14.6 cm^3^) (*p* > .05) (Figure 4).

### 3.2 Viscosity

**FIGURE 5:**
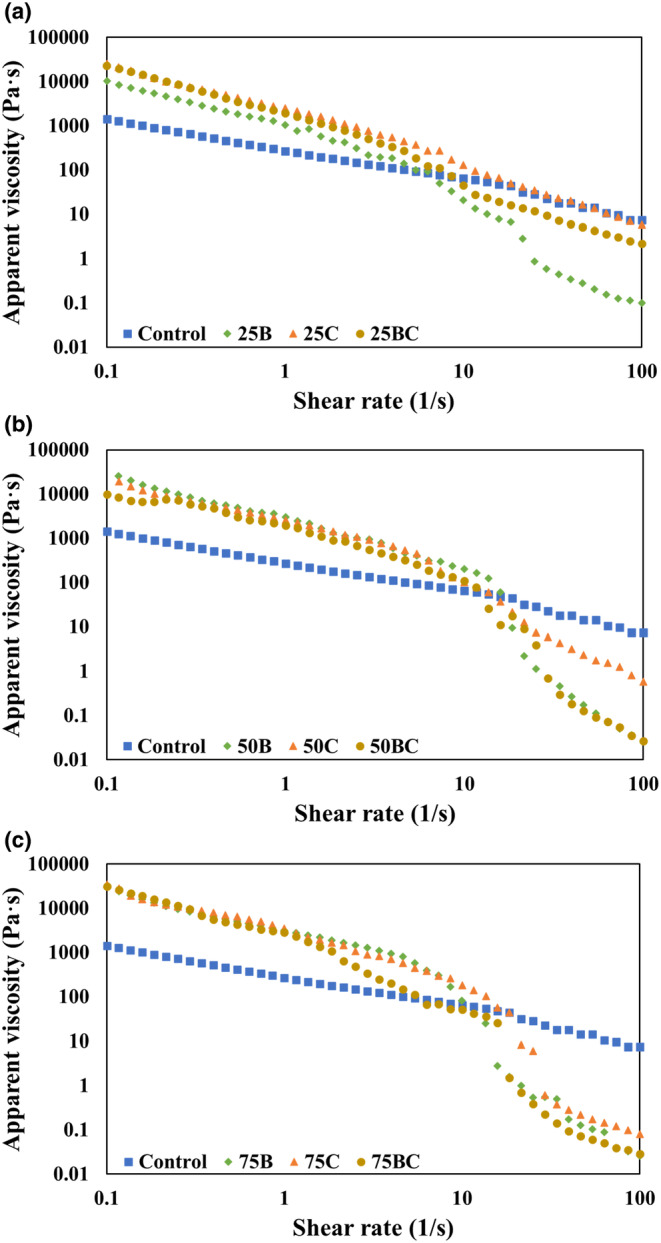
Apparent viscosities of the food inks with (a) 25:75, (b) 50:50, and (c) 75:25 vegetable:flour ratios, respectively.

Figure 5a–c depicts the viscosity of food inks prepared with 25%, 50%, and 75% (w/w) vegetable powders, respectively. The addition of different vegetable powders significantly increased the viscosity of the ink when compared to the control at low shear rates. The printing inks demonstrated a decrease in viscosity as the shear rate increased, confirming the presence of interactions that can be broken by the application of shear stress. The shear‐thinning, or pseudoplastic, behavior is correlated with the ability of inks to be easily extruded during 3D printing (Jiang et al., 2019) because the force required to print the ink decreases as shear is introduced, causing the ink to flow smoothly through the nozzle. The viscosity curves for the samples containing vegetables were similar (Figure 5). However, the results showed that broccoli had a greater effect on viscosity than carrots at shear rates >10 1/s. Specifically, 25BC, 50BC, and 75BC exhibited lower viscosities at shear rates higher than 10 1/s when compared to their carrot‐only counterparts. This is likely due to the higher particulate structure in the broccoli samples compared to that of carrots. This could explain the decreased printing accuracy with increasing broccoli concentrations in the ink formulation (50B, and 75B; Figure 4). It has been reported that adding carrot powder to wheat flour significantly increases the system's water absorption capacity, which could be attributed to an increase in fiber content as a result of the addition of or increasing the level of carrot powder. Our findings revealed that the broccoli‐containing samples required more water to reach a certain consistency than their carrot‐containing counterparts, which could be explained by broccoli's higher fiber content (Ying et al., 2021). Dried broccoli particles have been shown to swell up to 7.6 times their original size when absorbing water. The rheological behavior of dough systems made with wheat flour is considerably influenced by this swelling capacity (Ahmad et al., 2016; Silva et al., 2012). When high‐volume fractions are added, the system behaves as a cellular material rather than a gelled matrix with dispersed particles. Particulate suspensions are well known to exhibit shear‐thinning behavior, as demonstrated by the results obtained in this study (Moelants et al., 2013; Sharma et al., 2017). The control sample, on the other hand, indicated a lower shear effect than the other samples and did not print well due to the high adhesiveness of the ink (Figure 5). The characterization of the flow behavior for the vegetable inks is consistent with food inks developed from spinach and kale leaf purees, where the purees also used in 3D food printing displayed a shear‐thinning behavior (Pant et al., 2023).

### 3.3 Microstructural properties

**FIGURE 6:**
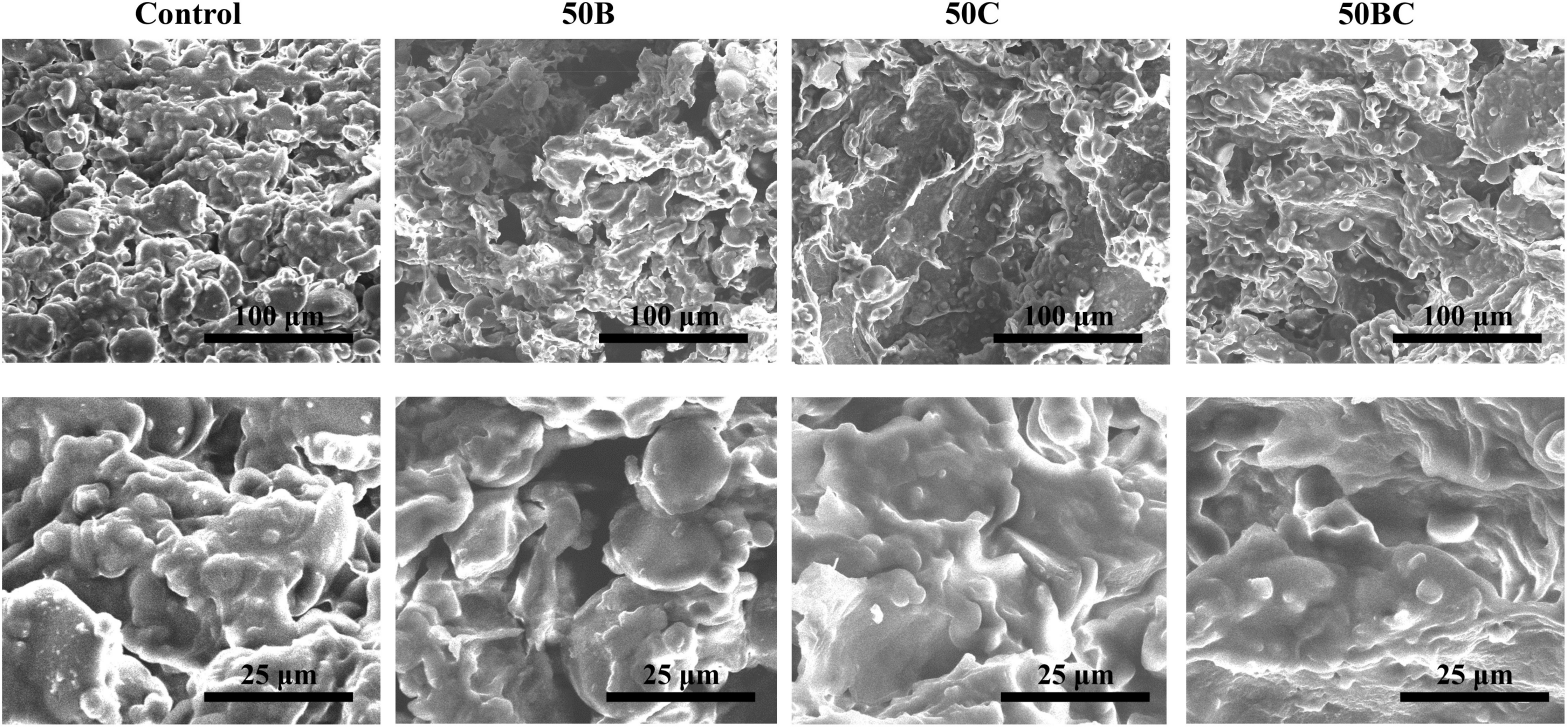
The SEM images taken from cross sections of the raw 3D‐printed snacks.

SEM images of the samples 3D‐printed with inks containing 50% carrot and/or broccoli are shown in Figure 6. The cross‐sectional structure of the samples revealed that the control sample made from wheat flour had a more granular structure compared to the samples containing vegetables. The wheat flour dough's microstructure included a protein (gluten) matrix and starch granules of varying sizes embedded into the protein matrix (Dahesh et al., 2016). The high elasticity of the gluten matrix in wheat flour dough caused the control sample to lose shape while printing. When the SEM images from 50B, 50C, and 50BC were compared to the control, noticeable differences in the microstructure of the inks were observed (Figure 6). According to the literature, fibers have a gluten dilution effect, resulting in a less porous structure in baked goods (Polaki et al., 2010). When 50% broccoli (50B) was added, an open structure was observed, showing that the gluten matrix became discontinuous and a number of starch granules leaked out, whereas 50% carrot (50C) and 50% carrot/broccoli (50BC) samples revealed a dense structure and starch granules are still connected to the gluten. It could be explained by the carrot's different ratio of soluble and insoluble fibers and their effect on the gluten matrix (Li et al., 2023). The observed morphological differences between the control and vegetable‐containing samples could lead to differences in snack‐quality characteristics.

### 3.4 Color analysis

**FIGURE 7:**
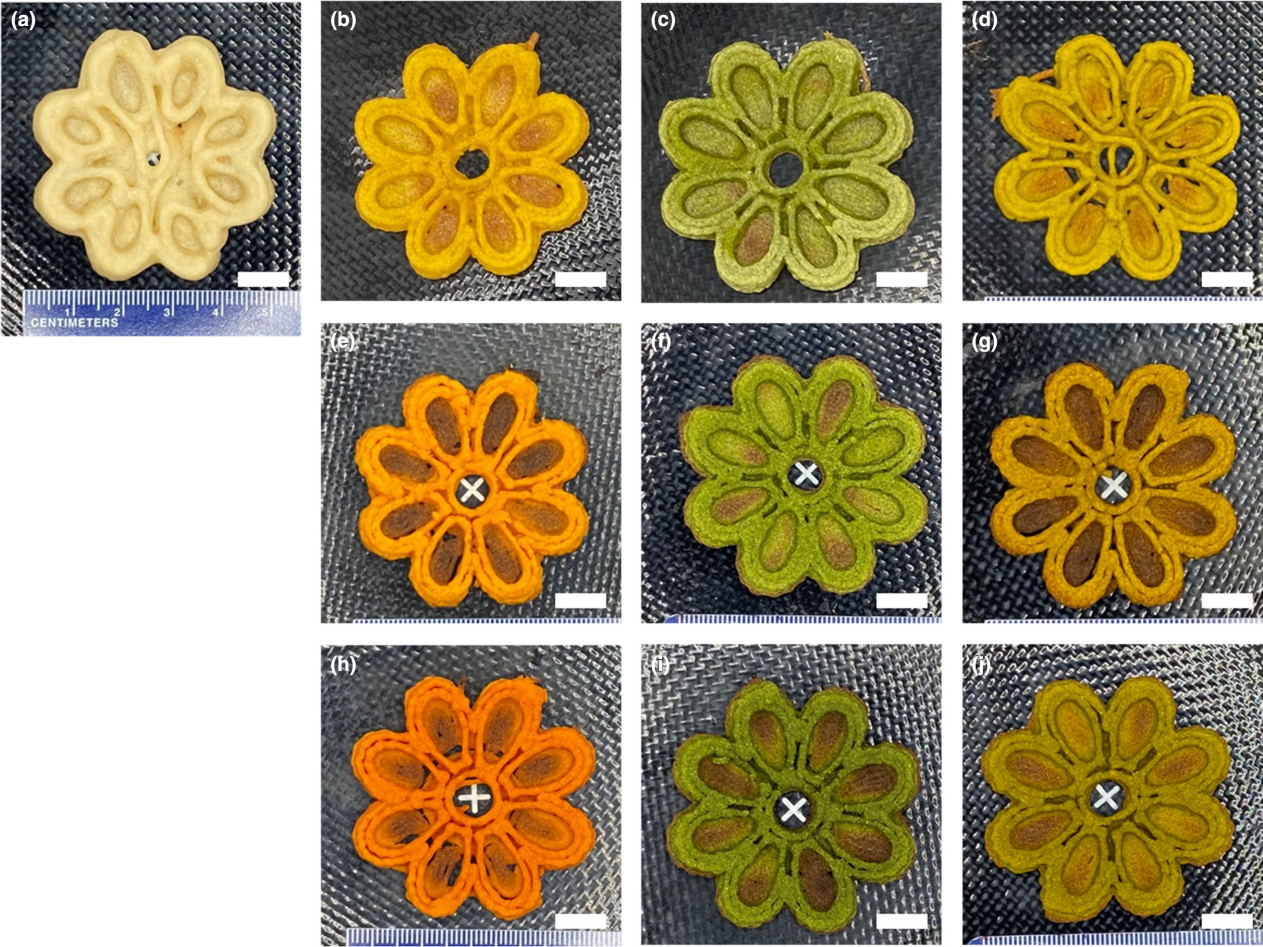
3D‐printed baked products of each food ink formulation (a: control; b: 25 carrot:75 flour; c: 25 broccoli:75 flour; d: 25 broccoli/carrot:75 flour; e: 50 carrot:50 flour; f: 50 broccoli:50 flour; g: 50 broccoli/carrot:50 flour; h: 75 carrot:25 flour; i: 75 broccoli:25 flour; j: 75 carrot/broccoli:25 flour, mass ratios). The scale bars in all images represent 1 cm.

Figure 7 depicts the pictures of the 3D‐printed snacks after baking, and Table 2 summarizes the color profile of the raw food inks and the cooked 3D‐printed snacks. Significant differences (*p* < .05) in the color parameters of the snacks with different formulations were noted. The snacks' color differences were complementary to the distinct appearances of their vegetable‐based substitutions.

**TABLE 2:** Color parameters of 3D‐printed snacks before and after baking.

	Raw			Baked		
	L*	a*	b*	L*	a*	b*
Control	90.41 ± 0.15^Aa^	4.35 ± 0.09 ^Ac^	10.23 ± 0.61^Af^	65.06 ± 0.28^Ba^	4.66 ± 0.06^Ac^	17.84 ± 0.23^Bg^
25C	75.17 ± 0.09^Ab^	23.38 ± 0.19^Ab^	79.12 ± 0.06^Aa^	61.15 ± 0.69^Bb^	16.20 ± 0.65^Bb^	66.65 ± 0.62^Ba^
25B	71.41 ± 0.84^Ac^	−12.42 ± 0.14^Ae^	29.45 ± 1.10^Ae^	53.21 ± 0.33^Bd^	−1.40 ± 0.50^Be^	23.66 ± 0.51^Bf^
25BC	71.33 ± 0.79^Ac^	1.10 ± 0.14^Ad^	61.94 ± 1.97^Ab^	56.38 ± 0.83^Bc^	3.57 ± 0.19^Bc^	52.57 ± 1.32^Bc^
50C	70.35 ± 0.50^Ac^	30.03 ± 0.34^Aa^	62.50 ± 1.21^Ab^	57.66 ± 0.20^Bc^	18.42 ± 0.23^Ba^	63.88 ± 0.15^Ab^
50B	64.34 ± 0.62^Ad^	−17.26 ± 0.62^Ag^	40.19 ± 1.22^Ad^	51.37 ± 1.47^Be^	−2.15 ± 0.12^Be^	26.49 ± 0.57^Bef^
50BC	65.00 ± 0.52^Ad^	1.35 ± 0.08^Ad^	60.07 ± 1.52^Ab^	54.16 ± 0.35^Bd^	3.55 ± 0.13^Bc^	42.45 ± 0.83^Bd^
75C	67.35 ± 0.18^Ae^	29.88 ± 0.37^Aa^	62.19 ± 0.63^Ab^	56.87 ± 0.45^Bc^	19.28 ± 0.74^Ba^	60.73 ± 1.67^Ab^
75B	59.82 ± 0.16^Af^	−15.09 ± 0.43^Af^	30.64 ± 1.11^Ae^	46.61 ± 0.48^Bf^	−3.74 ± 0.16^Bf^	15.69 ± 0.57^Bg^
75BC	60.53 ± 0.51^Af^	0.30 ± 0.33^Ad^	49.28 ± 1.86^Ac^	50.05 ± 0.44^Be^	0.32 ± 0.46^Ad^	28.40 ± 1.13^Be^

*Note*: Means with different capital letters within the same row and color parameter and means with different lowercase letters within the same column are significantly different (*p* < .05). Data are given as the means ± standard deviations.

Among both uncooked and baked snacks, the control had the highest lightness (90.41 and 65.06, respectively), as expected, followed by carrot‐incorporated snacks. The samples demonstrated corresponding decreases in lightness values after baking, indicating slight color changes during baking. The value for redness, “a*,” was highest in uncooked carrot‐containing snacks and decreased significantly after cooking (*p* < .05). Because of the higher yellowness values “b*,” the overall color of carrot‐incorporated snacks was orange. The uncooked broccoli snacks had a green color, as evidenced by the negative “a*” value, indicating a green shade. These values showed that the produced snacks had colors close to the respective vegetables used. Overall, the colors of the baked samples were less intense (Table 2), which could be due to pigment conversion. However, after baking, all the snacks had an acceptable, pleasing color.

### 3.5 Texture analysis

**FIGURE 8:**
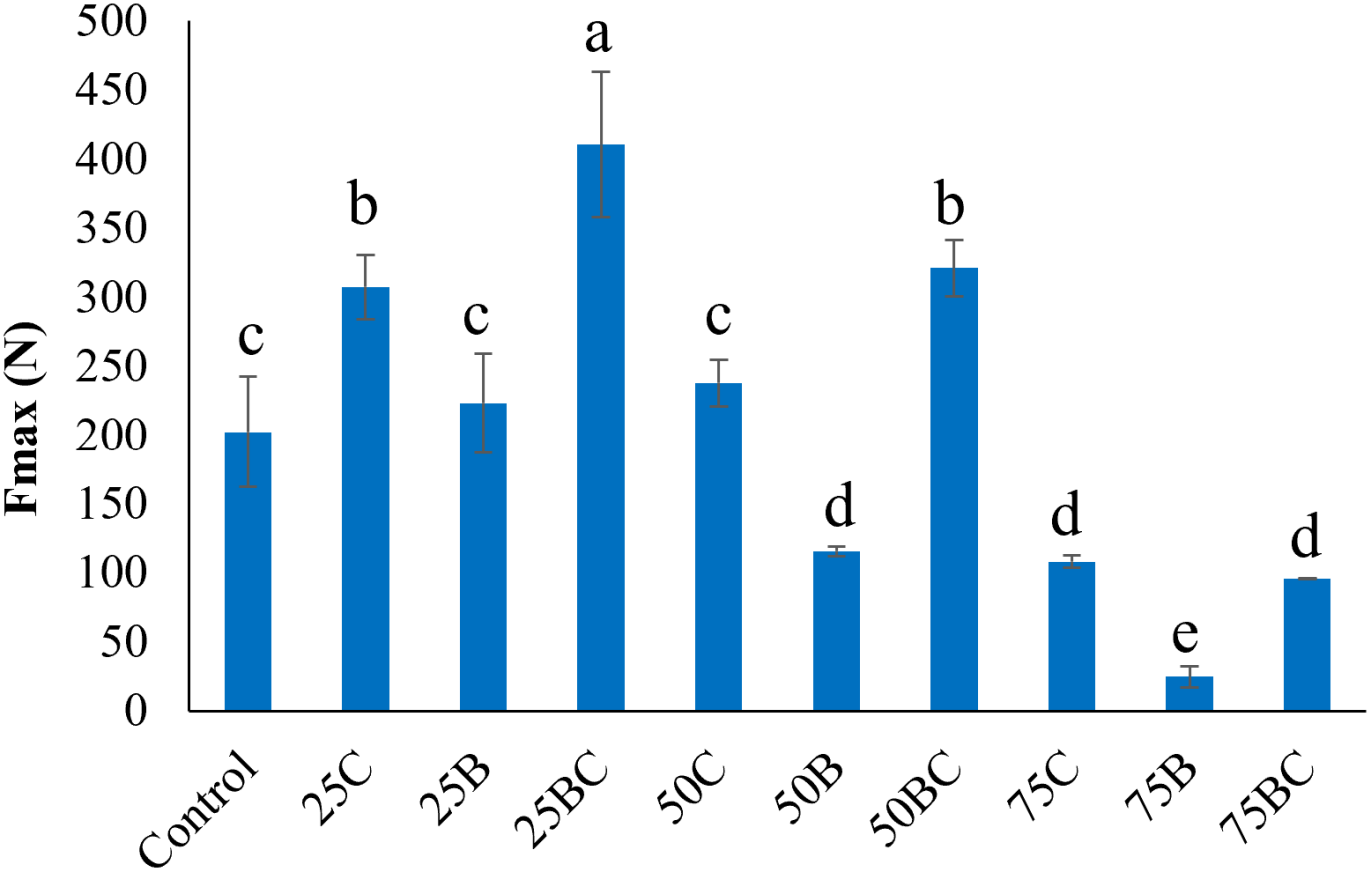
The hardness of the 3D‐printed samples after baking. Means with different superscript letters are significantly different (*p* < .05).

Textural properties are important factors in determining product quality as they affect human perceptions of texture. As shown in Figure 8, the maximum force from the compression test was determined and reported as the hardness of baked snacks. This value reflects the initial bite that a consumer would take of a food sample (Han et al., 2017). If the hardness of the snacks exceeds an optimal threshold, the flavor might be impacted, resulting in a diminished level of crispiness. When compared to the control sample, adding <75% carrot powder increased the strength of the ink and the hardness of the snack, while increasing the carrot amount to 75% decreased the hardness. The same trend was observed in broccoli‐containing samples by increasing their ratio. However, there was no significant difference in hardness between the 25B and control samples (*p* > .05). 50B and 75B indicated significantly lower hardness compared to the control (*p* < .05). In addition, carrot‐based snacks were considerably harder than broccoli‐containing snacks (Figure 8).

The water‐insoluble proteins in wheat flour are responsible for the rheological and structural properties of the dough, including elasticity and structural strength, as well as viscosity and fluidity. Dough made from wheat flour is a soft gel with a unique network structure that is viscoelastic and extensible. According to the literature, soluble dietary fibers effectively absorb water and wrap starch granules distributed across the gluten network structure. This function prevents many protein molecules from getting tightly entangled, preventing the formation of a cohesive spatial network. Furthermore, the addition of soluble dietary fibers to flour dilutes the gluten protein, affecting the formation of the gluten matrix (Jia et al., 2020). For broccoli‐containing samples, the snacks' hardness decreased, probably due to the reduced gluten network structure. Additionally, when broccoli was added to the snacks, higher porosity and open structure were observed, as demonstrated in SEM images (Figure 5), resulting in lower hardness. This correlation between porosity and hardness has also been observed in biscuits in a previous study (Umesha et al., 2015).

The difference between carrots and broccoli can be attributed to how their soluble and insoluble fiber contents affect the gluten matrix. According to the literature, both forms of fiber can interact with gluten proteins via various mechanisms, where the chemical reactivity of soluble and insoluble fibers is important in gluten protein aggregation (Zhou et al., 2021). Certain soluble fibers, such as pectin, are more reactive. This increased reactivity could be attributed to the number and accessibility of functional groups involved in the interaction with gluten proteins and water. Insoluble fibers having lower reactivity, such as cellulose, appear to be incorporated into the structure as fillers or physical barriers, which primarily exhibit steric effects (Zhou et al., 2021). Jia et al. (2020) investigated the effects of soluble dietary fiber on the physical properties of biscuits. They performed the texture profile analysis to analyze the texture of the products and found that adding soluble fibers reduced the hardness of the biscuits, which is consistent with the results we observed after adding broccoli and increasing the vegetable ratios in the snacks. Furthermore, the hardness (~50–450 N) measured in our study for vegetable snacks is comparable to the hardness (~84–350 N) reported for 3D‐printed cereal‐based snacks developed by Derossi et al.( 2021), where they 3D‐printed wheat flour‐based parallelepiped‐shaped objects (Derossi et al., 2021).

## 4 CONCLUSIONS

The loss of nearly one‐third of the world's vegetables, despite their sound nutritional value, could be reduced by finding a way to utilize the vegetables that have an imperfect appearance. 3D food printing technology is capable of changing the appearance of ugly vegetables into unique snack products. In this study, imperfect broccoli and carrots were freeze‐dried and then turned into food‐grade inks suitable for 3D printing applications. All the inks showed shear‐thinning behavior, making them ideal for extrusion‐based 3D food printing. The control sample had a low degree of shape integrity. As more vegetable powders were added, the printability of the inks improved. Samples containing 25% vegetable powder revealed noticeable lines, indicating inferior resolution. On the other hand, samples with 75% vegetable powder flooded together to produce a more homogenous appearance. The volume of the sample printed with 75% carrot/broccoli flour was comparable to the volume of the 3D digital model (14.3 cm^3^ vs. 14.6 cm^3^). Although there was a significant difference in color between raw and cooked samples, cooked samples still exhibited an appealing color. Morphological observations demonstrated a difference in the microstructure of control and vegetable‐containing samples, corroborating the variations in snack quality parameters, including hardness. In conclusion, imperfect vegetables originally considered food waste can be successfully upcycled to produce a 3D‐printed snack ideal for consumption.

## AUTHOR CONTRIBUTIONS

**Safoura Ahmadzadeh:** Formal analysis (equal); investigation (equal); methodology (equal); visualization (equal); writing – original draft (equal). **Taylor Clary:** Formal analysis (equal); investigation (equal); methodology (equal); visualization (equal); writing – original draft (equal). **Alex Rosales:** Formal analysis (supporting); methodology (supporting). **Ali Ubeyitogullari:** Conceptualization (lead); funding acquisition (lead); investigation (lead); methodology (lead); project administration (lead); resources (equal); supervision (equal); writing – review and editing (lead).

## CONFLICT OF INTEREST STATEMENT

There are no conflicts of interest to declare.

## ETHICS STATEMENT

This study does not involve any human or animal testing.

## Data Availability

Data sharing is not applicable to this article, as no new data were created or analyzed in this study.
